# Identification of a circadian-based prognostic signature predicting cancer-associated fibroblasts infiltration and immunotherapy response in bladder cancer

**DOI:** 10.18632/aging.206088

**Published:** 2024-08-30

**Authors:** Li Zhou, Jiaming He, Zhiming Hu, Hongwei Li, Jinlong Li

**Affiliations:** 1 Institute of Biotherapy, School of Laboratory Medicine and Biotechnology, Southern Medical University, Guangzhou, Guangdong, China; 2 Institute of Interdisciplinary Research, Guangdong Polytechnic Normal University, Guangzhou, Guangdong, China; 3 Research Institute of Guangdong Polytechnic Normal University in Heyuan City, Guangdong, China

**Keywords:** bladder cancer, circadian rhythm, cancer-associated fibroblasts, prognostic model, immunotherapy

## Abstract

Circadian rhythm disruption impacts the efficiency of both chemotherapy and immunotherapy, yet identifying the key factors involved remains challenging. Circadian rhythm disruption can trigger aberrant fibroblasts activation, suggesting potential roles of cancer-associated fibroblasts (CAFs) in addressing this issue. In this paper, TCGA-BLCA patients were classified into two subgroups based on the expression of core circadian rhythm genes (CCRGs). The CCRG-based subgroups showed distinct fibroblast-related signals, from which a risk model composed of five fibroblast-related genes was finally established with excellent survival prognostic value in both TCGA and GEO datasets. The risk model was positively associated with the infiltration of CAFs and can efficiently predict the immunotherapy response in BLCA. Besides, high-risk score was associated with reduced sensitivity to a majority of traditional chemotherapeutic drugs such as oxaliplatin and gemcitabine. Further, the correlation between CCRGs and the risk genes was analyzed. Among the five risk genes, *FAM20C* displayed the most extensive correlation with the CCRGs and exhibited the strongest connection with CAFs infiltration. Moreover, *FAM20C* independently served as a predictor for the response to immunotherapy in BLCA. In conclusion, this study has identified a circadian-based signature for evaluating CAFs infiltration and predicting the efficacy of chemotherapy and immunotherapy. The central gene *FAM20C* has emerged as a promising candidate which merits further investigations.

## INTRODUCTION

Bladder cancer (BLCA) is among the most common genitourinary malignancies with high morbidity and mortality. In 2023, it is estimated to account for 82,290 new cases and 16,710 deaths, constituting 2.7% of all cancer-related fatalities in the United States [1]. New immunotherapies utilizing immune checkpoint blockade (ICB), such as monoclonal antibodies targeting programmed death-1 (PD-1) and programmed death ligand-1 (PD-L1), have shown promise in BLCA treatment [2]. However, ICB has demonstrated limited success in BLCA, except for specific molecular subtypes with exceptional immunogenicity [3]. Additionally, the efficacy of ICB has reached a plateau, with the proportion of patients exhibiting durable clinical benefit showing no significant change over the years [4]. Therefore, it is crucial to identify additional prognostic markers or immune-enhancing strategies that could enhance the efficacy of this immune-stimulating treatment.

The circadian rhythm serves as a central regulator governing the essential activities of the body. Epidemiological and experimental evidences indicate that disruption of circadian rhythm facilitates tumor initiation and progression [5]. Conversely, reinforcement of biological rhythms may hinder cancer progression and reduce recurrence [5]. Many fundamental physiology aspects, including the immune system, are governed by circadian rhythms. The circadian rhythm regulates immune cell homing, migration and hence plays critical roles in adaptive immune responses [6]. Therefore, the circadian rhythm has long been pursued to enhance the outcomes of cancer treatment. Intriguingly, non-pharmaceutical interventions involving circadian-compliant timing of ICB administration have been associated with improved outcomes [4]. A study involving 146 patients with advanced melanoma revealed that ICB infusions administrated after 4:30 pm were associated with shorter overall survival, highlighting the advantages of morning over evening administration [7]. Similar findings were observed in a study on metastatic non-small cell lung cancer, demonstrating a remarkable four to five-fold increase in efficacy with morning ICB administration [8]. Therefore, the circadian rhythm presents a promising target for improving the efficiency of cancer therapy. However, due to the widespread impact or foundations of circadian rhythms, identifying the critical cues involved remains challenging.

Cancer-associated fibroblasts (CAFs) are the main stromal cells in tumor microenvironment (TME) that are integral to cancer malignant progression [9]. The functions of CAFs encompass a wide spectrum, including matrix deposition and remodeling, reciprocal signaling interactions with cancer cells, as well as crosstalk with infiltrating leukocytes. This makes them a potential target for optimizing therapeutic strategies against cancer [10]. Accumulating evidences indicate that circadian rhythm disruption leads to abnormal activation of fibroblasts and eventually resulting in fibrosis in various organs [11, 12]. In the context of CAFs, disruption of the circadian rhythm by genetic deletion method in mouse can promote transition of CAFs into myofibroblasts, thus exacerbating fibrotic phenotype and metastasis in various tumors [13]. Conversely, CAFs also have potential to regulate the circadian rhythm in tumor cells. Co-culture with CAFs can modify the oscillated expression of the circadian proteins in cancer cells [14]. Therefore, circadian rhythm disorder is closely related to the activation and function of fibroblasts, forming a mutually reinforcing process that promotes the malignant phenotype of tumors.

Hence, the purpose of this study was to develop a fibroblast-related signature based on the dysregulated circadian rhythm genes to predict prognosis and characterize the immune landscape of BLCA patients. In addition, the correlation between the circadian rhythm genes and the signature genes were analyzed to explore the possible regulatory networks.

## MATERIALS AND METHODS

### Data acquisition and processing

The bulk RNA-sequencing (RNA-Seq) data along with clinical information were retrieved from the TCGA-BLCA database (https://portal.gdc.cancer.gov). The 24 core circadian rhythm genes (CCRGs), namely *ARNTL*, *ARNTL2*, *CLOCK*, *CRY1*, *CRY2*, *PER1*, *PER2*, *PER3*, *TIMELESS*, *BHLHE41*, *BHLHE40*, *CSNK1D*, *CSNK1E*, *DBP*, *FBXL3*, *HLF*, *NFIL3*, *NPAS2*, *NR1D1*, *NR1D2*, *RORA*, *RORB*, *RORC*, and *TEF*, were sourced from previously published literature [15, 16]. Different expression of these genes between normal and tumor bladder tissue was analyzed using the “edgeR” package, with thresholds set at |log2FC| > 1.0 and *p* < 0.05.

Furthermore, the RNA expression data of BLCA, including GSE32894 (308 urothelial carcinomas), GSE13507 (165 primary bladder cancer patients), GSE149582 (12 de novo muscle-invasive bladder cancer and 14 progressive muscle-invasive bladder cancer samples), GSE128192 (28 cases of sarcomatoid carcinomas and 84 cases of conventional urothelial carcinomas), and the immunotherapy data GSE67501 (11 renal cell carcinoma samples from patients receiving anti-PD-1 immunotherapy), GSE78220 (28 melanoma samples from patients receiving anti-PD-1 immunotherapy), GSE35640 (65 melanoma samples from patients receiving MAGE-A3 immunotherapy), were obtained from the Gene Expression Omnibus (GEO) database. The BLCA immunotherapy data IMvigor210 (298 metastatic urothelial cancer samples from patients receiving PD-L1 blockade immunotherapy) study were acquired from the website (http://research-pub.gene.com/IMvigor210CoreBiologies/).

### BLCA subtype classification

The differentially expressed CCRGs between low- and high-stage of BLCA were utilized to subtype patients through the unsupervised clustering analysis using the “ConsensusClusterPlus”R package. The K-means clustering algorithm was used to identify stable circadian-based subgroups within BLCA. Since k = 2 is sufficient for well-separated subgroups, the patients were separated into subgroup 1 and subgroup 2 (C1 and C2). Subsequently, differentially expressed genes (DEGs) between the circadian-based subgroups were analyzed using the “edgeR” package, with thresholds set at |log2FC| > 1.0 and FDR < 0.01. Then, Gene Ontology (GO) enrichment analysis and Kyoto Encyclopedia of Genes and Genomes (KEGG) pathway analysis were performed using the “clusterProfiler” R package to uncover the functional roles of the DEGs between the C1 and C2 rhythm-related subgroups.

### Construction and validation of risk model

DEGs between the circadian-based subgroups implicated in the fibroblast-related signals were subjected to univariate Cox regression analysis to identify prognostic genes. Subsequently, the selected genes underwent further screening via Lasso and multivariate Cox regression analysis to establish a prognostic model.

To validate the risk model, patients were categorized into high- and low-risk groups based on the median risk score. The overall survival (OS) was then compared between the two groups using the “survminer” R package. The prediction accuracy of the signature was assessed by constructing a time-dependent receiver operating characteristic (ROC) curve using the “survival” R package. Additionally, “FactoMineR” package was used to perform principal component analysis (PCA). Furthermore, a nomogram, integrating clinical characteristics and the risk score, was created using the “rms” R package to estimate the probability of OS in BLCA at 1, 3, and 5 years.

### Gene set enrichment analysis (GSEA) of the risk model

GSEA analysis between the high- and low-risk groups was carried out using the GSEA 4.3.2 software. Discrepancies in pathways were analyzed using the Canonical Pathways gene sets (CP) which derived from the KEGG, Pathway Interaction Database (PID) and Reactome pathway databases. Variations in cell types were analyzed using the Curated Cancer Cell Atlas (3CA) metaprograms gene sets which consist of genes that are coordinately upregulated in subpopulations of cells within tumor [17], as well as the Cell Type Signature gene sets containing curated cluster markers for cell types identified in single-cell sequencing studies. Furthermore, specific genes were analyzed using the Oncogenic Signature gene sets and Transcription Factor Targets gene sets (TFT) [18, 19].

### Tumor stromal score and immune landscape evaluation

The stromal score and immune landscapes of TCGA-BLCA were assessed using a variety of algorithms, including the ESTIMATE [20], CIBERSORT [21], EPIC [22], XCELLp [23], MCPCounter [24], QUANTISEQ [25] and TIMER [26]. Additionally, Tumor Immune Dysfunction and Exclusion (TIDE) scores of TCGA-BLCA were downloaded from the official website http://tide.dfci.harvard.edu/download/. Differences of the stromal score, immune landscapes and checkpoint genes expression between the high- and low-risk groups were analyzed by the “limma” R package.

### Drug sensitivity prediction

The Genomics of Drug Sensitivity in Cancer 2 (GDSC2) database were downloaded from the GDSC website (https://www.cancerrxgene.org/). Drug sensitivity of each BLCA patient was estimated using the “oncoPredict” R package [27]. The “oncoPredict” algorithm used the GDSC2 expression matrix and drug response data as training data to compute the inhibitory concentration (IC_50_) value for 198 chemotherapeutic agents, based on the RNA expression data from the samples provided by the users.

### Bioinformatic platforms

The mutation and CNV alteration patterns of the 24 core CCRGs in the TCGA-BLCA were analyzed via the cBioPortal platform (https://www.cbioportal.org). Correlation of *FAM20C, TBX1* with exhausted T-cell and effector Treg cells was analyzed using the GEPIA2 (http://gepia2.cancer-pku.cn). The correlated expression as well as the Kaplan-Meier curves of *FAM20C, TBX1* gene with CAFs were analyzed through the TIMER2 (http://timer.comp-genomics.org/timer/). The correlation of *FAM20C* mRNA expression with OS in BLCA patients (including bladder [28, 29] and urothelial cancers [30] receiving ICB immunotherapy) was analyzed through the Kaplan-Meier plotter (https://kmplot.com).

### Statistical analysis

R software version 4.2.2 (R Foundation for Statistical Computing, Vienna, Austria) (http://www.R-project.org) was used for data analysis. Continuous variables were presented as mean ± standard deviations and compared through Wilcoxon test. Cox proportional hazard model was applied to the independent prognostic factors analysis. Violin diagrams and boxplots were drawn by the “ggplot2” and “ggpubr” R packages. The correlated expression of the risk genes with the CCRGs was analyzed by the “corrplot” R packages. Statistical significance is defined as *p < 0.05, **p < 0.01, and ***p < 0.001.

## RESULTS

### Dysregulation of circadian rhythm genes in BLCA tissues

**Figure 1:**
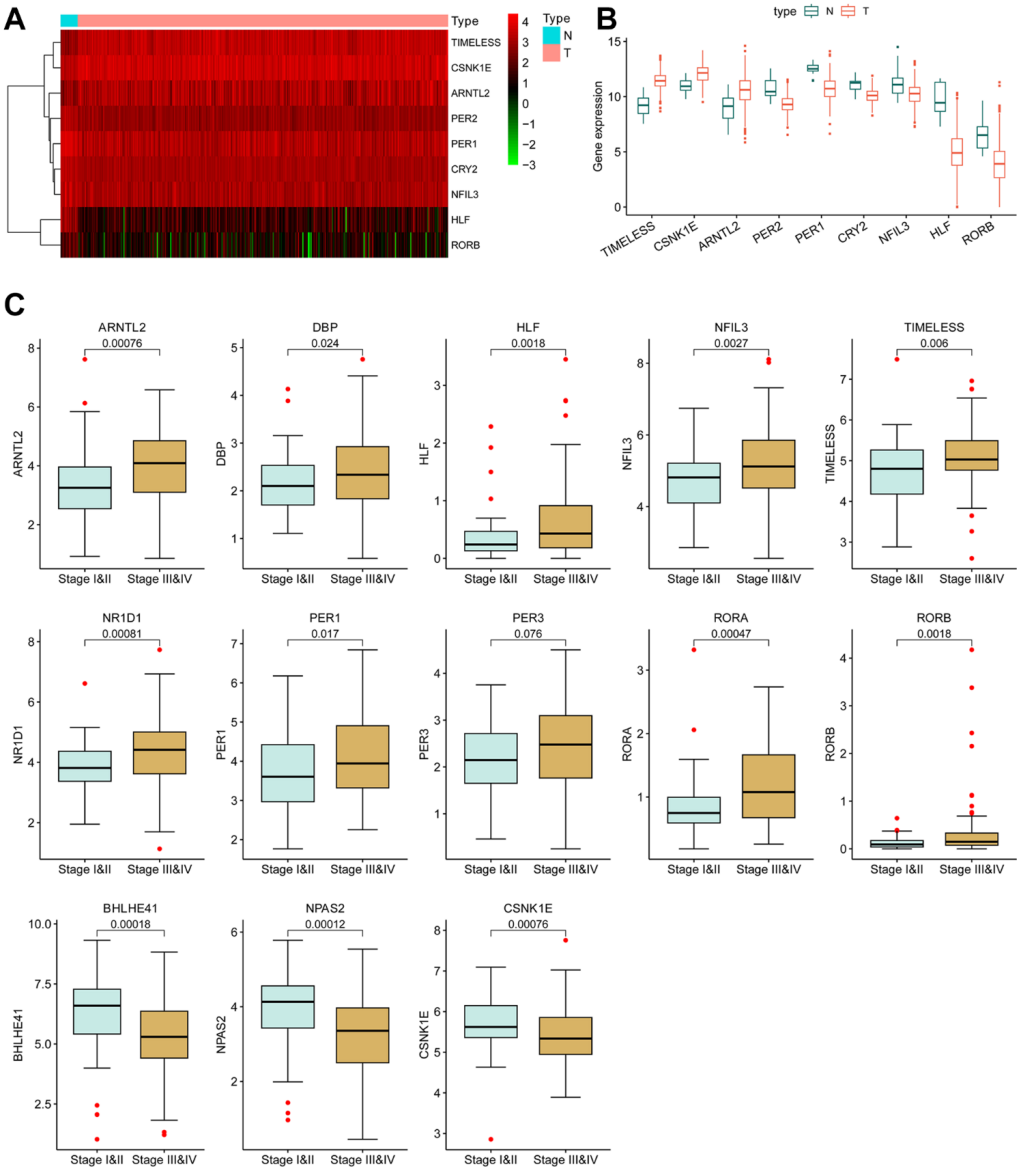
**Differentially expressed circadian rhythm genes in BCLA.** (**A**) Heatmap and (**B**) boxplot of the differentially expressed CCRGs mRNAs between BLCA tumors and normal tissues. (**C**) The correlation of CCRGs mRNAs with tumor stages.

The mutation rate of most CCRGs in bladder cancer tissues was less than 2%. Likewise, the frequency of copy number variation (CNV) alterations is also low, with the majority being lower than 4% (Supplementary Figure 1). Given the low frequent genetic alterations, our focus shifted to the differentially expressed mRNA. Among the 24 CCRGs, three genes were found to be up-regulated and five genes were down-regulated in tumors compared to normal tissues (Figure 1A, 1B).

Furthermore, the dysregulated expression of CCRGs was observed to be correlated with tumor stage. In comparison to low-stage tumors (stage I and II), ten CCRGs were up-regulated and three CCRGs were down-regulated in the high-stage tumors (stage III and IV) (Figure 1C). These findings suggest a potential contribution of CCRGs to the malignant progression of BLCA.

### BLCA sub-clustering based on CCRGs expression

**Figure 2:**
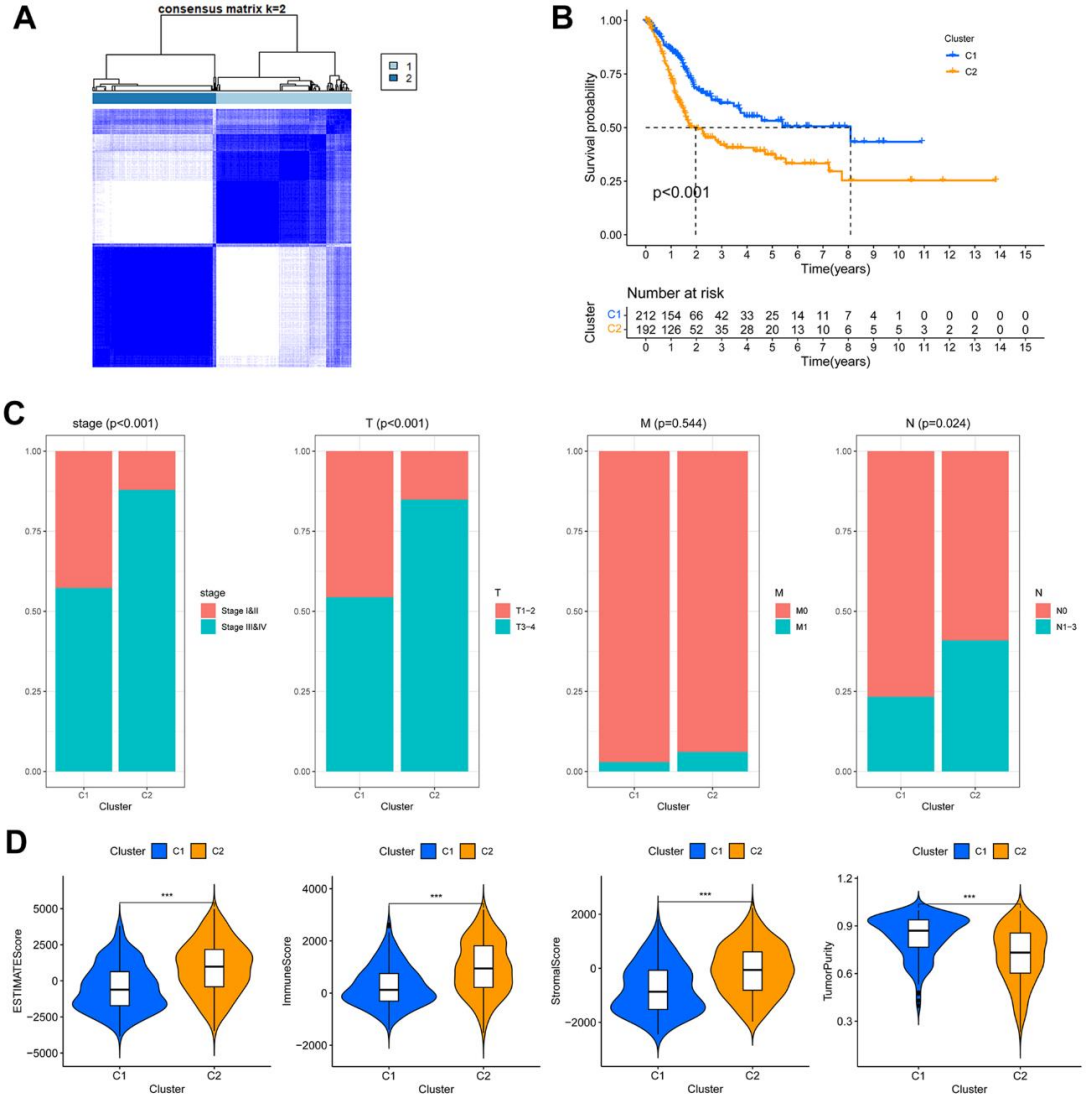
**The circadian rhythm-based subgroups of TCGA-BLCA.** (**A**) The TCGA-BLCA was divided into two circadian rhythm-based subgroups via unsupervised clustering. (**B**) Kaplan-Meier analysis of the OS of C1 and C2 subgroups. (**C**) Association of the circadian rhythm-related subgroups with stage and pathological T/M/N stages. (**D**) The ESTIMATE, stromal, immune score and tumor purity of the two circadian rhythm-related subgroups, *** *p* < 0.001.

The circadian clock exerts biological functions through regulating target genes expression. Therefore, the differentially expressed CCRGs may give rise to subgroups of tumors with distinct gene expression profiles. Based on the 13 differentially expressed CCRGs between low- and high-stage tumors (Figure 1C), the TCGA-BLCA patients were clustered into two subgroups (C1 and C2) (Figure 2A). C2 subgroup exhibited significantly worse OS compared to C1 (*P* <0.001, Figure 2B). In addition, the circadian-related subgroups were associated with clinicopathological features. The C2 subgroup included more patients in higher tumor stages (stage III and IV) and higher pathological T and N stage (Figure 2C). Moreover, the C2 subgroup demonstrated notably higher ESTIMATE, immune and stromal scores, but lower tumor purity (Figure 2D). These results suggested that the CCRGs- based BLCA subgroups have distinct TME profiles and prognostic outcomes.

A substantial number (3426) of differentially expressed genes (DEGs) were found between the C1 and C2 subgroup, with 2410 being up-regulated and 1016 being down-regulated in the C2 subgroup (Supplementary Figure 2A, 2B). GO analysis of these DEGs revealed a significant enrichment in epidermis development, intermediate filament organization, and keratinization, indicating differing cellular phenotypes between the two subgroups. Furthermore, GO terms related to TME constitution and immune cell infiltration, such as leukocyte migration, collagen-containing extracellular matrix, and receptor-ligand activity, were highly enriched in the C2 subgroup (Supplementary Figure 2C, 2D). Likewise, KEGG analysis also demonstrated a high enrichment of pathways relevant to TME constitution in the C2 subgroup, such as ligand-receptor interaction, cytokine-cytokine receptor interaction, Calcium signaling pathway and cell adhesion molecules, etc. (Supplementary Figure 2E). Together, these results indicated that the circadian-based C2 subgroup exhibits a highly active TME and poor prognosis.

### Construction of a risk signature based on the different fibroblasts-related signals between the circadian-based subgroups

**Figure 3:**
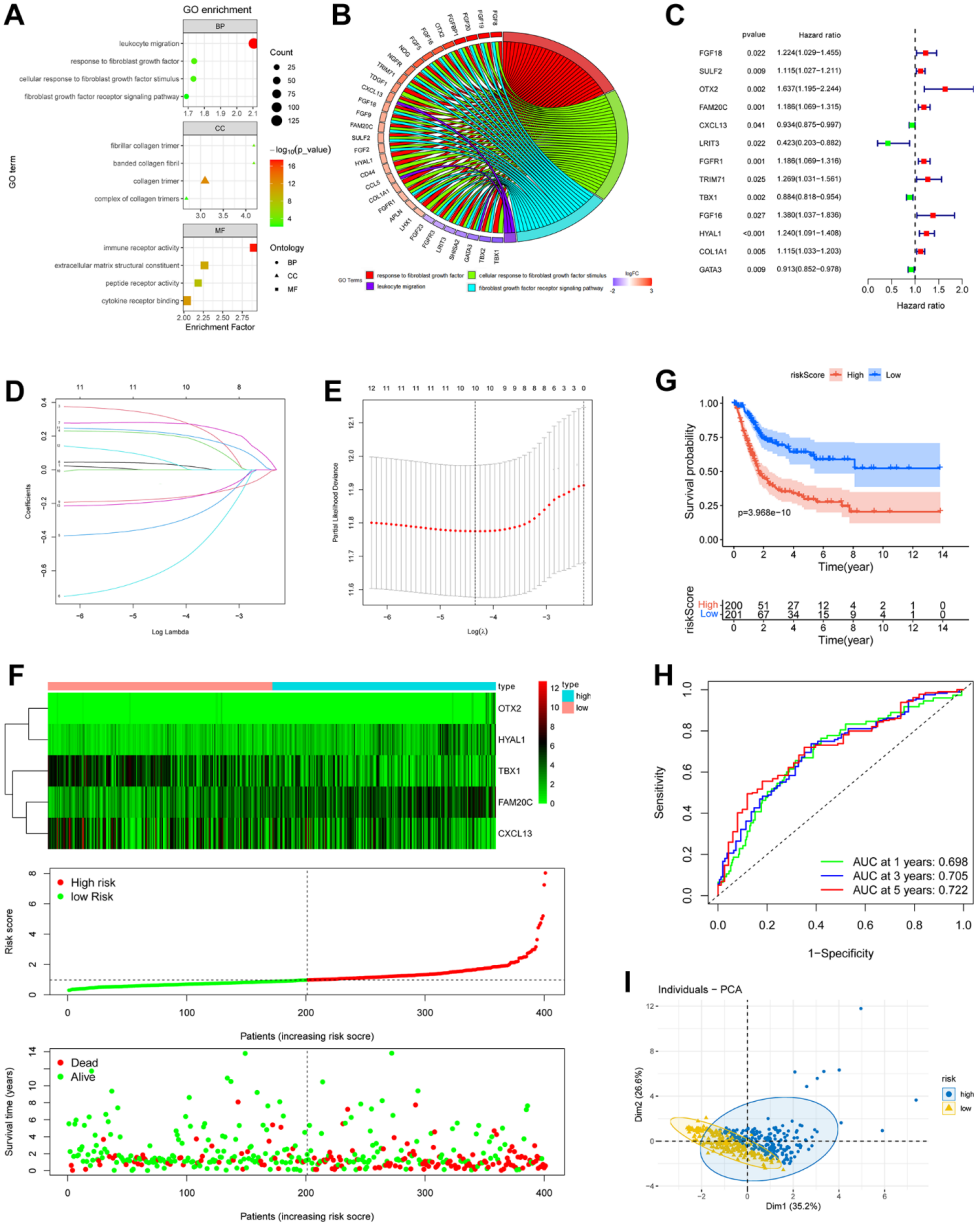
**Construction of risk model based on fibroblast growth signal difference.** (**A**) Bubble plot of GO enrichment of fibroblasts-related DEGs between C1 and C2 subgroups. (**B**) GO chord diagram showed the potential biological processes of the DEGs. (**C**) Forest plot of the survival-related DEGs obtained by univariate Cox regression, *p* < 0.05. (**D**, **E**) The 13 prognostic DRGs were fed into Lasso regression model. (**F**) Expression profiles of the risk genes (up), distribution of risk score (middle) and survival status (down) of BLCA patients. (**G**) Kaplan-Meier survival analysis of BLCA patients based on risk score. (**H**) ROC curves demonstrated the predictive prognostic value of risk score at 1, 3 and 5 years. (**I**) PCA analysis map of the high- and low-risk groups.

In the Biological Processes (BP) of GO, the fibroblast growth factor signals and leukocyte migration process were highly enriched in the DEGs between C1 and C2 subgroup (Figure 3A). Consistently, in the Cellular Component (CC) and Molecular Function (MF) categories, collagen- and immune receptor-related terms were highly enriched (Figure 3A). Considering the pivotal role of fibroblasts in the immunosuppressive tumor microenvironment [31], we consequently directed our attention to the DEGs implicated in the fibroblast growth factor signals and leukocyte migration process. These DEGs were further selected based on their enrichment in more than one biological process (Figure 3B). Ultimately, 31 DEGs were chosen for further investigation, and among them, 13 genes were identified as holding prognostic value (Figure 3C). After Lasso (Figure 3D, 3E) and multivariate Cox regression analysis, 5 genes (*OTX2, FAM20C, CXCL13, TBX1, HYAL1*) were ultimately selected to construct a risk model. The risk score was calculated based on the mRNA level and the weights of the selected genes as follows:

Risk score = (0.4806) × *OTX2* + (0.2076) × *FAM20C* + (−0.1431) × *CXCL13* + (−0.1049) × *TBX1* + (0.1385) × *HYAL1*.

BLCA patients were then stratified into high- and low- risk groups according to the median risk score. A significant worse prognosis was observed along with gradually increasing risk score (Figure 3F). Kaplan-Meier analysis revealed a notably lower overall survival rate in high-risk patients (Figure 3G). The ROC curves demonstrated that the area under the curve (AUC) of the risk score at 1, 3, and 5 years were 0.698, 0.705, and 0.722, respectively (Figure 3H). In addition, the PCA analysis illustrated a clear separation of the high-risk group from the low-risk group along dimension 1 (Figure 3I).

### Validation of the risk model

**Figure 4:**
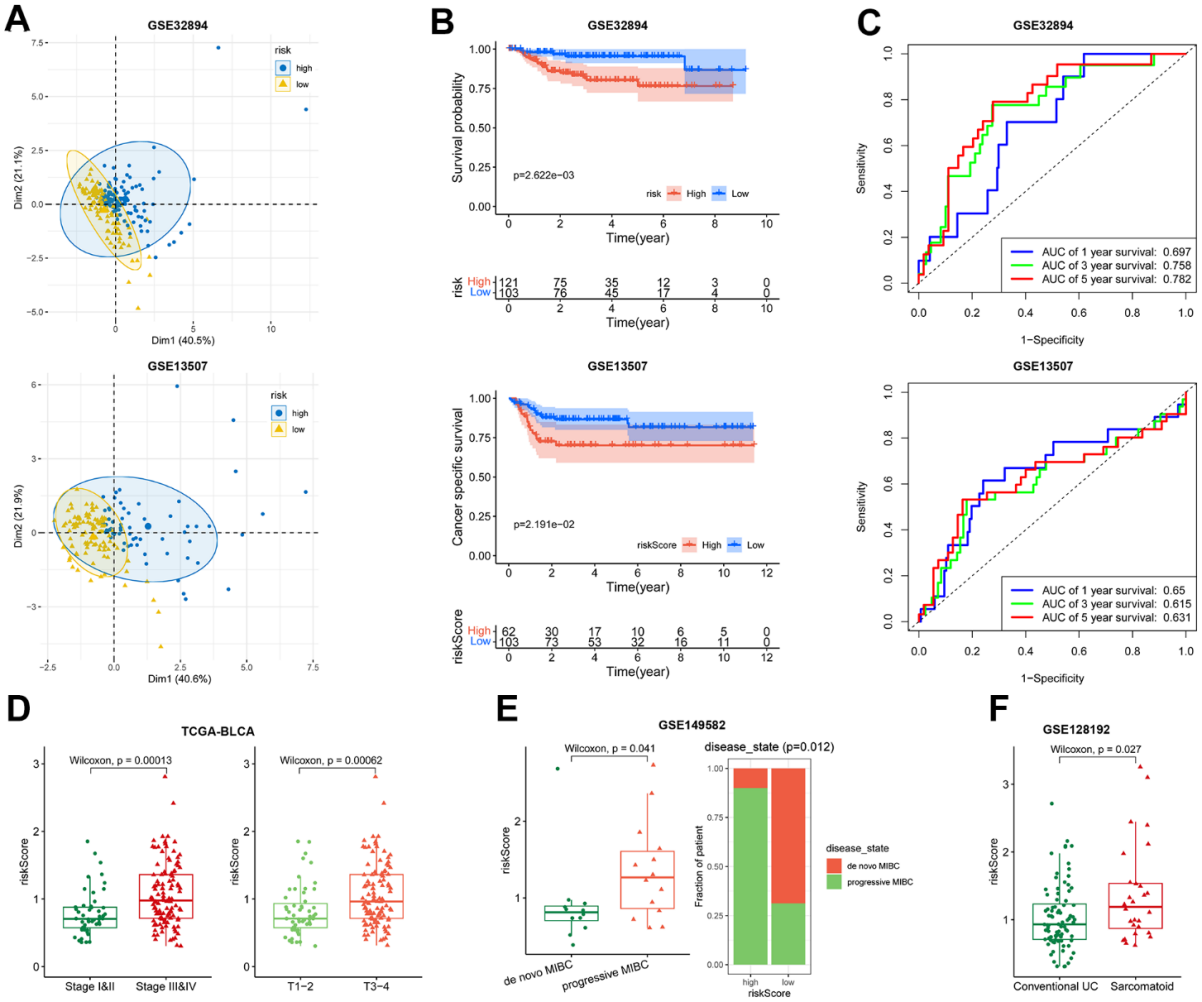
**Association of the risk model with overall survival and disease severity.** (**A**–**C**) The PCA analysis map, Kaplan-Meier survival analysis, and ROC curves of the high- and low-risk patients in GSE32894 (up) and GSE13507 (down) datasets. (**D**) Plot of risk score according to the stage (left) and T (right) in TCGA-BLCA dataset. (**E**) Patient’s risk score in de novo MIBC vs. progressive MIBC (left), and composition of the two disease states in high- and low-risk patients (right) in GSE149582 dataset. (**F**) Patient’s risk score in conventional UC vs. sarcomatoid carcinoma in GSE128192 dataset.

Performance of the 5-gene risk model was validated in GSE32894 (n = 254) and GSE13507 (n = 164) BLCA cohorts. In both cohorts, PCA analysis demonstrated satisfactory separation of the two risk groups (Figure 4A). High-risk patients displayed worse survival probability compared with low-risk patients (Figure 4B). the AUC for predicting 1, 3 and 5-year overall survival were 0.697, 0.758, 0.782 in the GSE32894 cohort, and 0.65, 0.615, 0.631 in the GSE13507 cohort (Figure 4C). These results underscore the prognostic capacity of the risk signature for BLCA.

The risk model was found to be linked with disease severity. In TCGA-BLCA, the risk score exhibited a positive correlation with tumor stage. High-stage tumors (stage III-IV, T3-T4) displayed higher risk scores than low-stage tumors (stage I-II, T1-T2) (Figure 4D). In GSE149582 (n = 26) dataset, the progressive muscle-invasive bladder cancer (MIBC), known for its heightened aggressiveness compared to de novo MIBC [32], displayed substantially higher risk scores (Figure 4E). In another BLCA cohort, GSE128192 (n = 112), the risk score of conventional urothelial carcinoma (UC) was less than that of sarcomatoid carcinoma (Figure 4F). The latter is characterized by a mesenchymal phenotype with a pronounced propensity for regional and distant metastasis [33]. Together, these results indicate that the risk signature offers insight into a more aggressive phenotype.

### Gene set enrichment analysis (GSEA) between high- and low-risk groups

**Figure 5:**
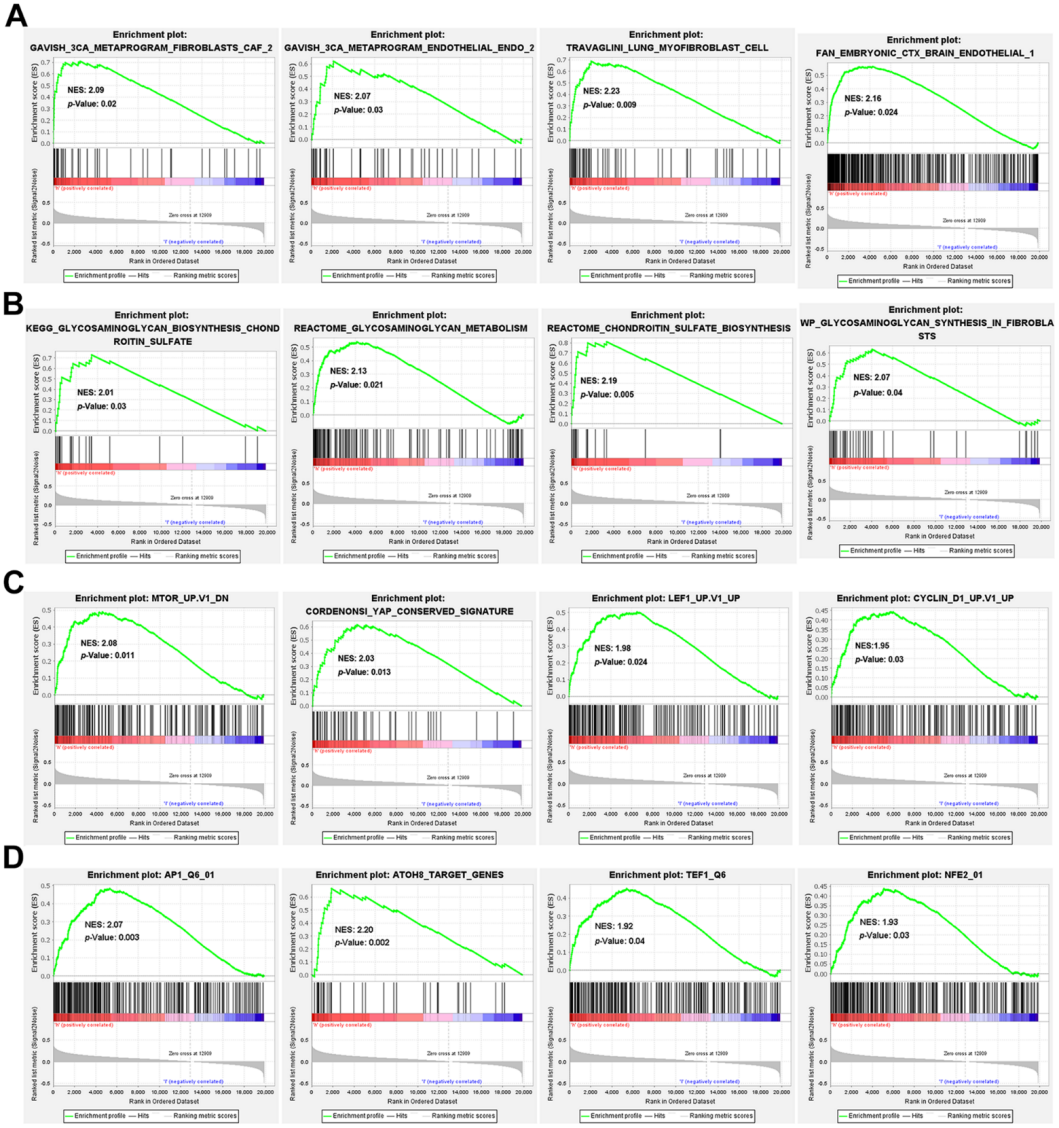
**GSEA enrichment between high- and low-risk groups.** GSEA enrichment based on (**A**) the 3CA metaprograms and the cell type signature gene sets, (**B**) the canonical pathways gene sets derived from the KEGG, Reactome and WikiPathways pathway database, (**C**) the oncogenic signature gene sets and (**D**) the transcription factor targets (TFT) gene sets.

Genes related to extracellular matrix, including matrix constituent, angiogenesis, ECM-receptor interaction, focal adhesion, integrin pathways and epithelial to mesenchymal transition were enriched in the high-risk group as shown in various gene sets (Supplementary Figure 3). The 3CA metaprograms and the single-cell sequencing gene sets unveiled elevated levels of fibroblasts and endothelial cells in the high-risk group (Figure 5A). Interestingly, the high-risk group exhibited heightened levels of glycosaminoglycan and chondroitin sulfate metabolism (Figure 5B). Further, in the oncogenic signature gene sets, mTOR, YAP, LEF1 and CYCLIN D1 were found to be activated in the high-risk group (Figure 5C). Finally, the TFT gene sets showed amplified levels of transcription factor potential for AP1, ATOH8, TEF1, and NFE2 within the high-risk group (Figure 5D).

### Association of the risk model with the immune landscape

**Figure 6:**
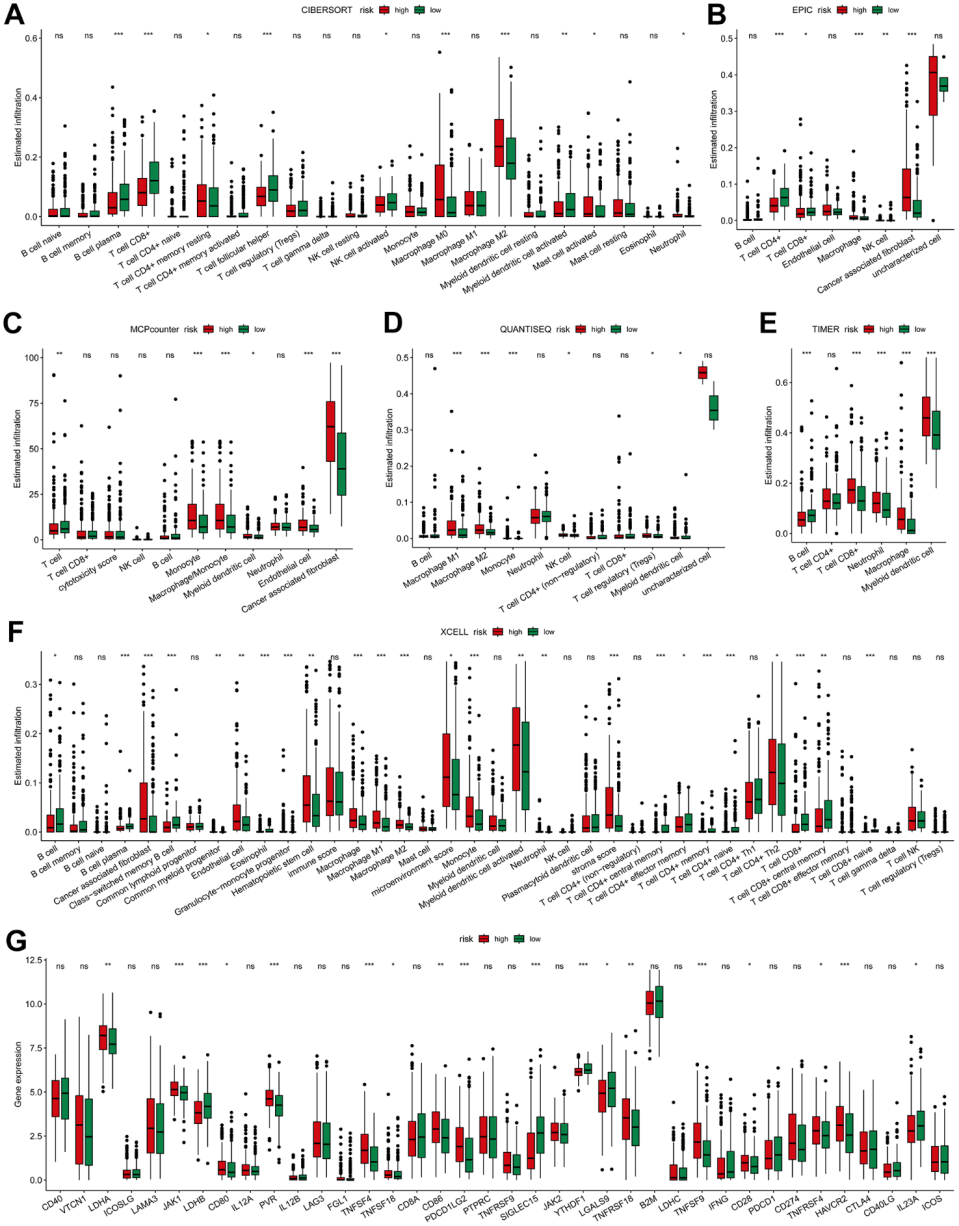
**Immune-related association of the risk model.** The difference of high- and low-risk groups in tumor immune landscape evaluated by (**A**) CIBERSORT, (**B**) EPIC, (**C**) MCP-Counter, (**D**) QUANTISEQ, (**E**) TIMER and (**F**) XCELL. (**G**) Differences in the immune checkpoint-related genes expression between high- and low-risk groups. * *p* < 0.05, ** *p* < 0.01, *** *p* < 0.001.

Immune landscape analysis using diverse algorithms revealed elevated infiltration of both macrophages (Figure 6A–6F) and CAFs (Figure 6B, 6C, 6F) in the high-risk group. The behavior of CD8^+^T cells exhibited varying trends depending on the specific algorithm employed. CIBERSORT, EPIC and XCELL indicated a decrease in CD8^+^ T cell infiltration in high-risk group (Figure 6A, 6B, 6F). In contrast, TIMER demonstrated increased CD8^+^T cell infiltration in the high-risk group (Figure 6E). Meanwhile, MCPCounter and QUANTISEQ indicated no significant difference in CD8^+^T cell infiltration between the two groups (Figure 6C, 6D). As for B cells, most algorithms suggested no discernible trend of change (Figure 6A–6D). Further, the expression of 38 routine immune checkpoint-related genes in high- and low-risk tumors was compared. Many of those genes, such as *HAVCR2, TNFSF9, TNFSF4, PDCD1LG2, CD86, PVR, JAK1*, etc., exhibited significant upregulation in high-risk tumors (*p* < 0.001). However, the most prominent immune checkpoint genes, *PDCD1* (*PD-1*), *CD274* (*PD-L1*), and *CTLA4*, showed no differential expression between the two groups (Figure 6G). These results indicate that high risk is associated with increased infiltration of CAFs and macrophages.

### The risk score predicts efficiency of BLCA immunotherapy and chemotherapy

**Figure 7:**
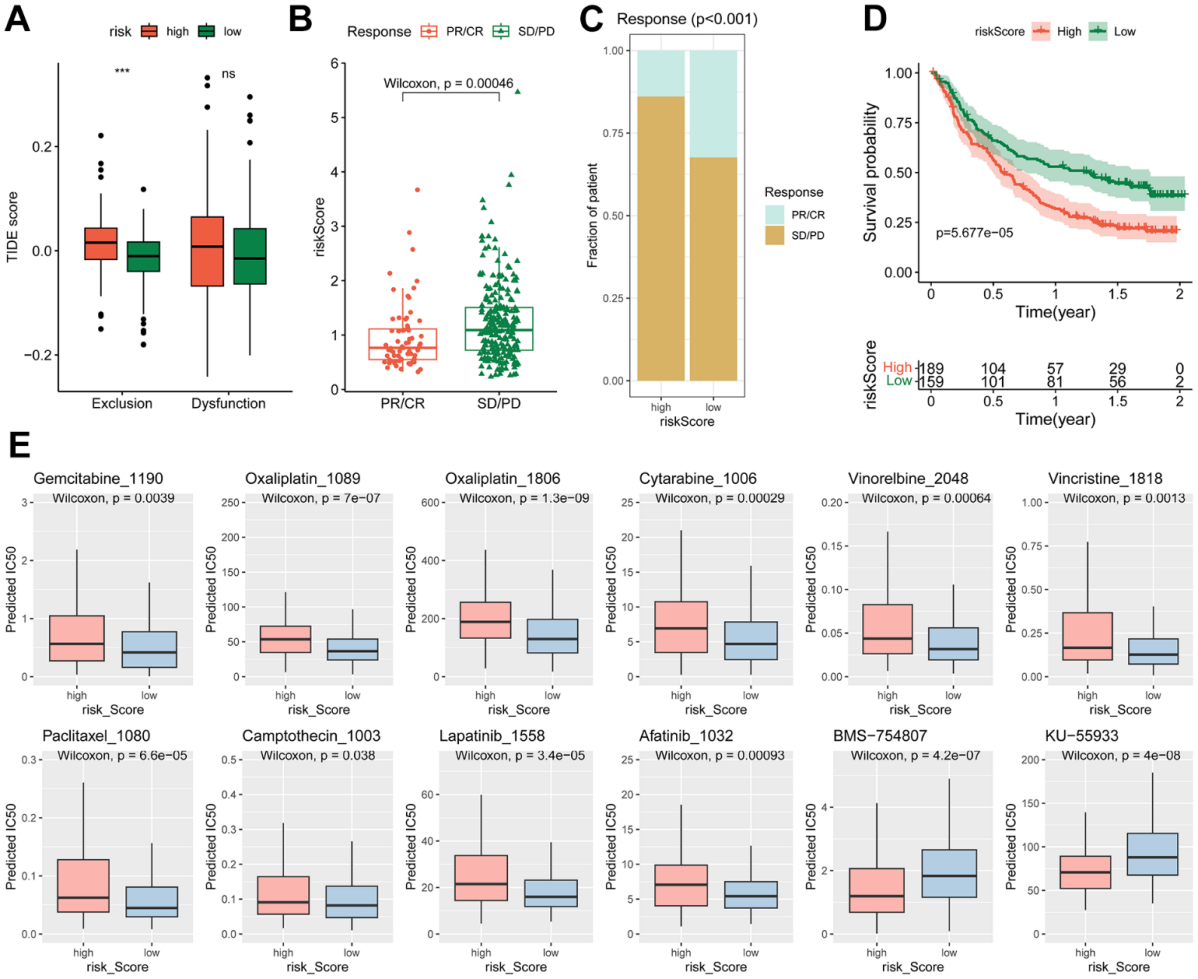
**Different immunotherapy response and chemotherapy sensitivity between high- and low-risk groups.** (**A**) Differences of the TIDE score between high- and low-risk groups, *** *p* < 0.001. (**B**–**D**) Relationship of risk score with immunotherapy efficiency in IMvigor210 cohort. (**B**) Difference of risk score in different response groups. (**C**) Distribution of different response in high- and low-risk groups. (**D**) Kaplan-Meier curve of the high- and low-risk patients. (**E**) The estimated IC_50_ of chemotherapy drugs.

Given that the TIDE score has the ability to predict cancer immunotherapy response [34], we then examined the correlation of the risk model with the TIDE scores. It showed that the high-risk group exhibited a higher T cell exclusion score (Figure 7A), while the T cell dysfunction score showed no significant difference between high- and low-risk groups. This is consistent with the elevated abundance of CAFs in high-risk group (Figure 6B–6D). As T cell exclusion is the major obstacle for efficient BLCA immunotherapy [30]. The risk score may hold predictive value for BLCA immunotherapy.

Subsequently, the predictive effect of risk score for immunotherapy was evaluated. In the Imvigor210CoreBiologies cohort, the non-response patients (SD/PD) had higher risk scores than the response patients (PR/CR) (Figure 7B, 7C). The high-risk group displayed significantly worse overall survival (OS) than the low-risk group (Figure 7D). However, in immunotherapy for other types of tumors, such as non-small cell lung carcinoma (GSE135222) and melanoma (GSE78220, GSE91061), the risk signature failed to predict outcomes (Supplementary Figure 4).

To test the potential value of the risk signature predicting drug sensitivity, the predicted IC_50_ of 198 drugs were analyzed by the “oncoPredict” R package. The findings revealed that the high-risk group exhibited reduced sensitivity to a majority of traditional chemotherapy drugs, including oxaliplatin, gemcitabine and cytarabine. Similarly, anti-mitotic agents such as vinorelbine, vincristine, paclitaxel, camptothecin, and certain targeted agents, such as lapatinib (an ErbB2 tyrosine kinase inhibitor) and afatinib (an EGFR inhibitor), displayed lower efficacy in the high-risk group. Conversely, certain types of inhibitors, such as BMS-754807 (an IGF1R inhibitor) and KU-55933 (an ATM inhibitor), were found to be more effective in the high-risk group (Figure 7E).

### Nomogram construction

**Figure 8:**
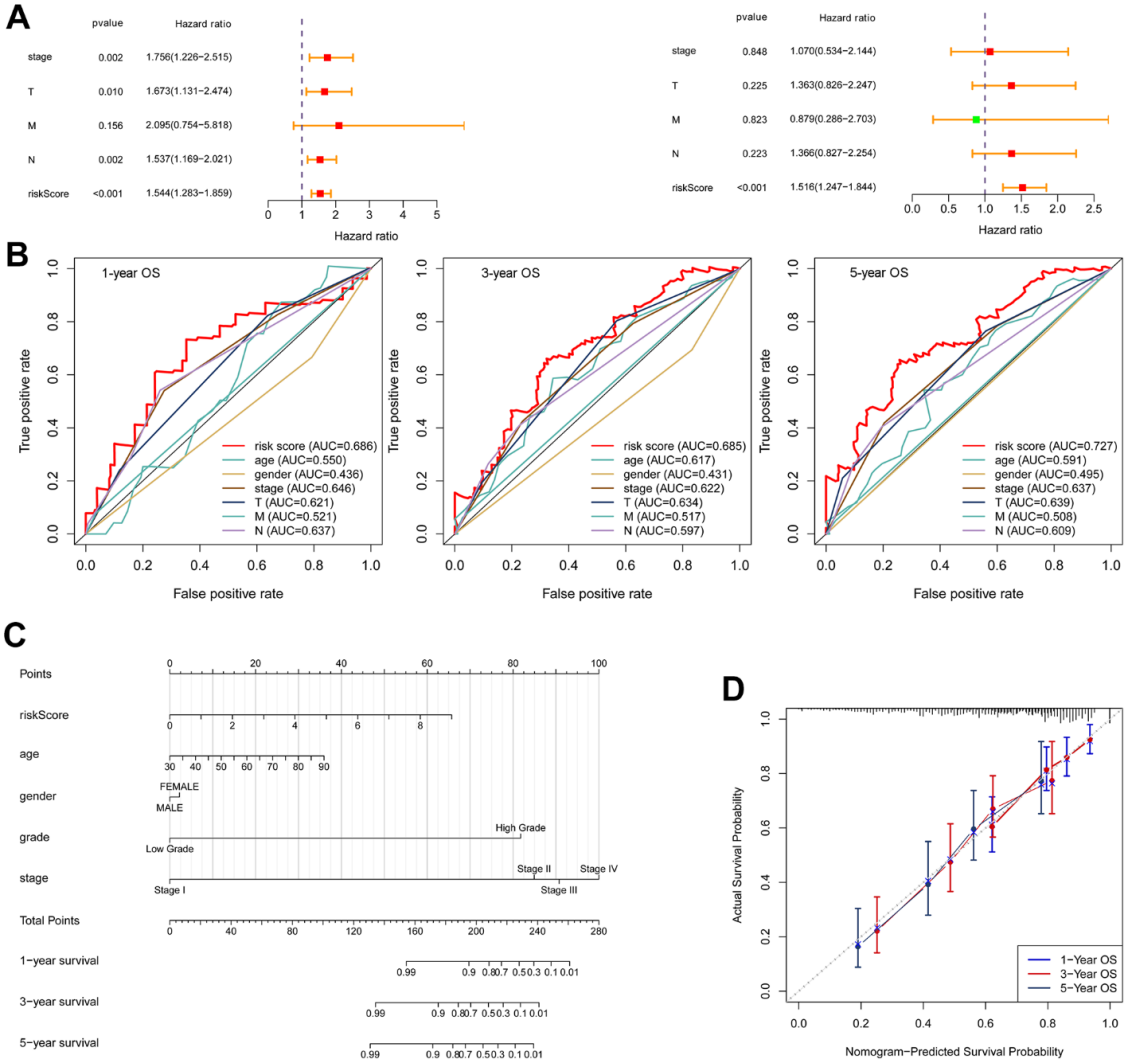
**Construction of a nomogram.** (**A**) Univariate Cox regression (left) and multivariate Cox regression (right) indicated the risk score as an independent prognostic factor. (**B**) ROC curves of the risk score and clinical characteristics. (**C**) Nomogram consisting of risk score and clinical factors. (**D**) Calibration curve for validation of the nomogram for estimating survival at 1-, 3- and 5-years.

The univariate and multivariate Cox regression analyses confirmed that the risk score was an independent prognostic factor for OS (Figure 8A). In multivariate ROC analysis, the risk score displayed a much more favorable performance than traditional prognostic factors in predicting 1-, 3-, 5-year OS (Figure 8B). To better forecast the prognosis of BLCA patients, a nomogram consisting of the risk score and clinical factors was constructed (Figure 8C). The calibration curve showed good performance for prediction of 1-, 3- and 5-year OS (Figure 8D).

### Correlated expression of the risk genes with the CCRGs

**Figure 9:**
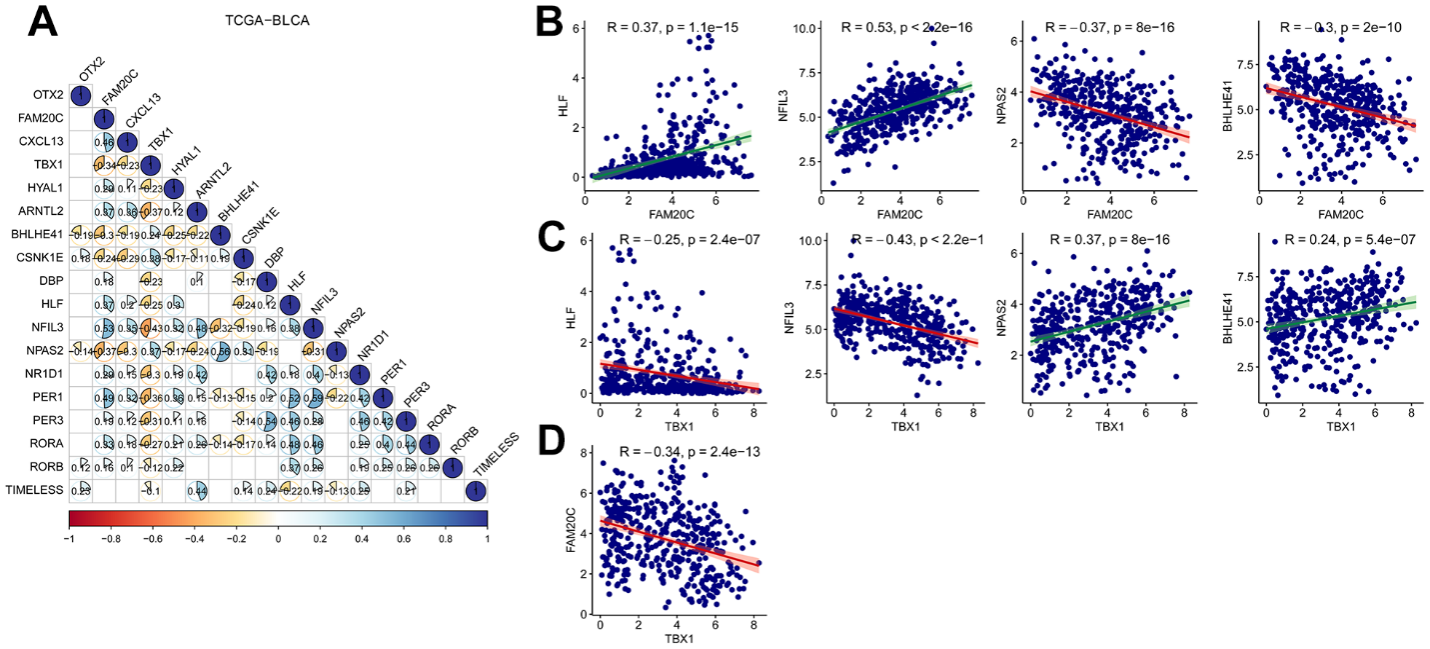
**Correlation analysis of risk genes with the CCRDs.** (**A**) Correlated expression of the risk genes with the CCRGs in TCGA-BLCA. (**B**) Correlated expression of *FAM20C* and (**C**) *TBX1* with *HLF, NFIL3, NPAS2* and *BHLHE41*. (**D**) *FAM20C* expression was negatively correlated with *TBX1*. Method = “Pearson”.

The risk signature was constructed based on the DEGs between CCRG-based subgroups. Therefore, expression of the risk genes is expected to be associated with the CCRGs. Then we analyzed the correlated expression of the risk genes with the CCRGs in TCGA-BLCA, GSE13507 and GSE32894 datasets. In the three datasets, the expression of risk genes was broadly correlated (positively and negatively) with the CCRGs (Figure 9A and Supplementary Figure 5A, 5B). Among the five risk genes, *FAM20C* and *TBX1* displayed the most extensive correlation with the CCRGs. Interestingly, the correlation patterns of *FAM20C* and *TBX1* with CCRG were exactly opposite. *FAM20C* was positively correlated with *HLF* and *NFIL3*, and negatively correlated with *NPAS2* and *BHLHE41*(Figure 9B). On the contrary, *TBX1* correlated with these CCRGs in the opposite direction to *FAM20C* (Figure 9C). Consistent with this result, a negative relationship between *FAM20C* and *TBX1* was observed (Figure 9D). Similar results were obtained in GSE13507 and GSE32894 datasets (Supplementary Figure 5C, 5D). These results indicated a regulatory network consisting of *FAM20C*, *TBX1* and the CCRGs.

### *FAM20C* and *TBX1* are associated with CAFs infiltration in opposite way

**Figure 10:**
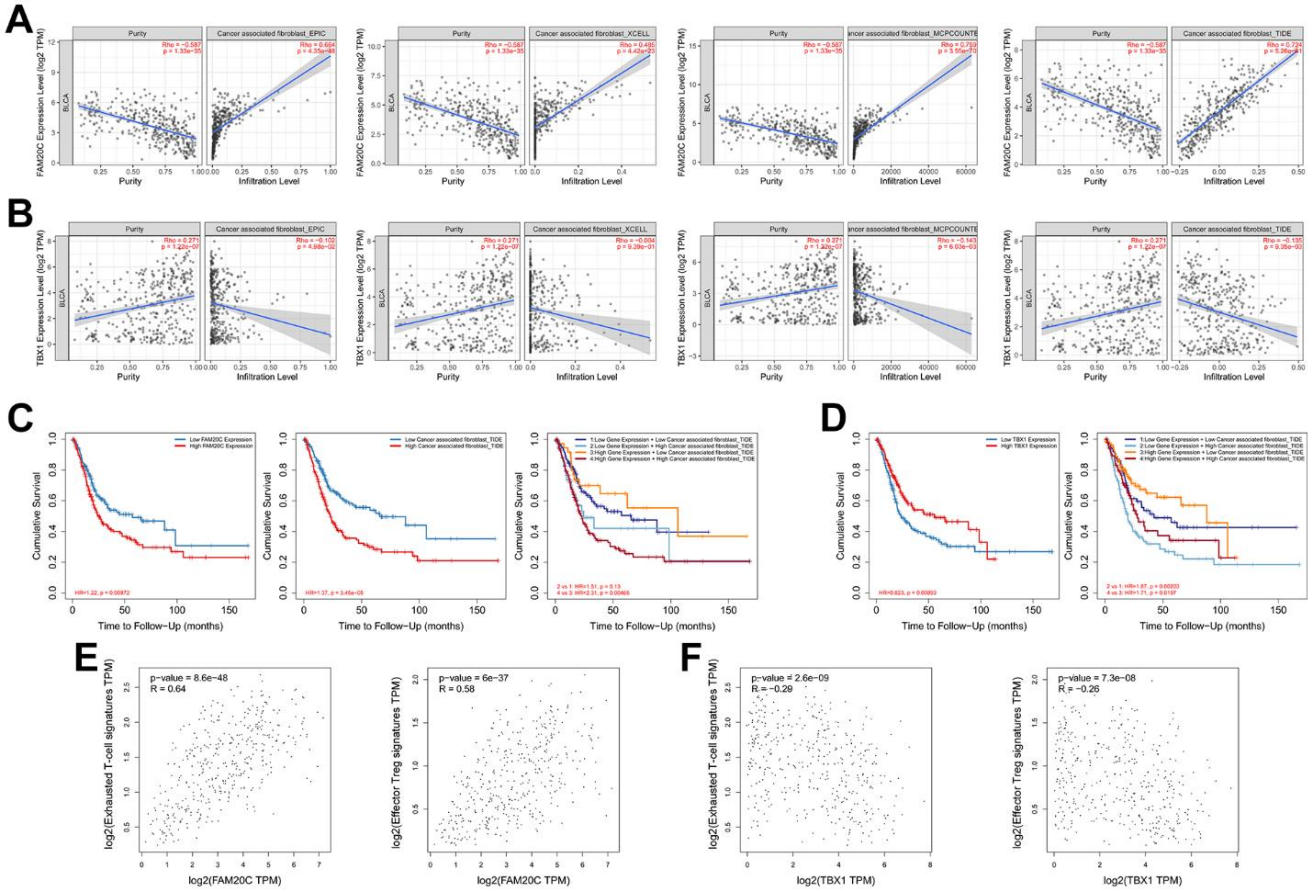
**Correlation analysis of FAM20C and TBX1 with the immunosuppressive microenvironment.** (**A**) Correlation of *FAM20C* or (**B**) *TBX1* expression with infiltration of CAFs. The CAFs were estimated by EPIC, XCELL, MCPcounter and TIDE. (**C**, **D**) Kaplan-Meier plot of different groups as indicated. (**E**) Correlation of *FAM20C* or (**F**) *TBX1* with exhausted T-cell and effector Treg cells. Exhausted T-cell signatures: *HAVCR2, TIGIT, LAG3, PDCD1, CXCL13, LAYN*; effector Treg cell signatures: *FOXP3, CTLA4, CCR8, TNFRSF9*.

To further identify the hub risk gene affecting the immunosuppressive microenvironment, we analyzed the correlation between the expression of individual risk genes and the infiltration of CAFs using the TIMER2. Among the five risk genes, *FAM20C* showed the strongest correlation with CAFs (Table 1). Multiple algorithms (EPIC, MCPcounter, TIDE and XCELL) affirmed a positive correlation between *FAM20C* and CAFs (Table 1 and Figure 10A). On the contrary, *TBX1* displayed a negative correlation with CAFs in EPIC, MCPcounter and TIDE (Table 1 and Figure 10B).

**Table 1:** Correlation of risk genes with CAFs infiltration in BLCA.

**Risk genes**		**Cancer associated fibroblasts (CAFs)**			
		**EPIC**	**MCPcounter**	**TIDE**	**XCELL**
*FAM20C*	rho (*p*-value)	0.66 (***)	0.76 (***)	0.72 (***)	0.48 (***)
*CXCL13*	rho (*p*-value)	0.17 (***)	0.21 (***)	0.13 (*)	0.13 (*)
*HYAL1*	rho (*p*-value)	0.19 (***)	0.24 (***)	0.22 (***)	0.17 (*)
*OTX2*	rho (*p*-value)	0.05 (0.59)	0.01 (0.93)	0.00 (0.99)	-0.02 (0.86)
*TBX1*	rho (*p*-value)	-0.10 (*)	-0.14 (**)	-0.14 (**)	0.00 (0.96)

*p < 0.05, **p < 0.01, ***p < 0.001.

Kaplan-Meier analysis revealed that high level of *FAM20C* or elevated CAFs could independently predict poor survival of BLCA patients. The coexistence of these two factors led to the most unfavorable survival outcomes. Conversely, the survival disadvantage associated with *FAM20C* was mitigated by low CAFs (Figure 10C). This highlights a collaborative function of FAM20C and CAFs in threatening survival. In contrast to *FAM20C*, *TBX1* was linked to a survival benefit, which was attenuated by high CAFs infiltration (Figure 10D).

The diametric association of *FAM20C* and *TBX1* with immunosuppressive microenvironment was further supported by their associations with exhausted T-cell and effector Treg cells. *FAM20C* was positively correlated with exhausted T-cell and effector Treg cells (Figure 10E). In contrast, *TBX1* exhibited a negative correlation with these cell types (Figure 10F). Together, these results suggest that FAM20C and TBX1 may exert opposing effects on the immunosuppressive microenvironment, with CAFs possibly being the target of action.

### *FAM20C* is an independent predictor for immunotherapy efficiency of BLCA

**Figure 11:**
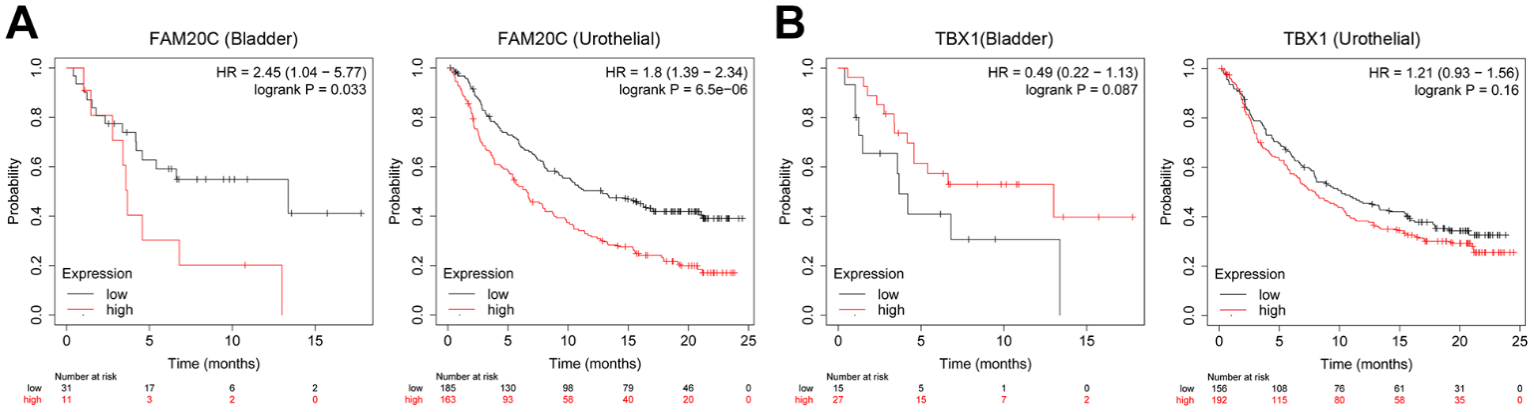
**Association of *FAM20C* and *TBX1* expression with immunotherapy efficiency.** (**A**) Survival analysis of the indicative genes in the immunotherapy of BLCA (anti-PD-1) and (**B**) urothelial carcinomas (anti-PD-L1) through the Kaplan-Meier plotter platform.

Given the association of *FAM20C* and *TBX1* with CAFs infiltration, we wonder whether they can predict the immunotherapy efficiency. We explore this issue through the Kaplan-Meier plotter. The results indicated that high *FAM20C* levels were indicative of poor survival in immunotherapy for bladder and urothelial carcinomas (Figure 11A). However, *TBX1* failed to predict the immunotherapy efficiency. No significant survival difference between high- and low-*TBX1* groups was observed (Figure 11B).

## DISCUSSION

CAFs play significant roles in the tumor microenvironment and have a considerable impact on the efficacy of cancer treatment. Through the production of cytokines, chemokines, and exosomes containing non-coding RNAs, CAFs induce resistance to diverse anticancer treatments, encompassing chemotherapy, radiotherapy, and immunotherapy [35, 36]. Moreover, the desmoplastic reaction induced by CAFs can diminish therapeutic efficacy by impeding drug delivery and infiltration of immune cells [36]. Specifically in the context of immunotherapy, CAFs contribute to the increased stiffness/rigidity of the extracellular matrix, thereby obstructing the infiltration of effector T cells, ultimately leading to immunotherapy resistance [37]. Accordingly, depletion of CAFs has been demonstrated to disrupt the structural integrity of TME, rendering treatment-resistant cancers susceptible to subsequent anti-PD-1 immunotherapy [38]. Therefore, targeting the aberrant activation of CAFs represents an efficacious strategy for both chemotherapy and immunotherapy [31]. However, the complexity and heterogeneity of CAFs pose challenges for therapeutic approaches in cancers [39].

Recent studies have demonstrated the enhancing impact of circadian rhythm disruption on the activation of fibroblasts [11]. The relevant studies have been predominantly focused on tissue fibrosis in a variety of organs, such as pulmonary fibrosis [40], atrial fibrillation and interstitial fibrosis in the heart blood vessel [41, 42], liver fibrosis [43, 44], kidney fibrosis [45], and adipose tissue fibrosis [46]. In these studies, distinct circadian genes, such as *NR1D1*(*Rev-Erbα*) [40, 43], *CLOCK* [42, 44], *BMAL1* and *CRY* [46] have been shown to regulate the transition of fibroblasts to myofibroblasts, the latter represents the typical activated form of fibroblasts. These studies confirmed the relationship between circadian rhythms disruption and fibroblasts behavior. However, limited research has been reported in the context of CAFs. A recent study utilizing a genetic deletion mouse model has revealed that the deletion of *BMAL1* exacerbates a fibrotic phenotype in colorectal, pancreatic and hepatocellular cancers [13]. Collectively, these findings underscore the significance of circadian rhythms in regulating fibroblasts activity.

Given the critical contributions of CAFs to the malignant process of cancers, several CAFs-related risk signatures have been reported in BLCA [47, 48]. Recently, by integrating scRNA-seq and bulk RNA sequencing datasets, an inflammatory CAFs-related signature [49] and an CAFs subclusters-related signature [50] have been established. These signatures have potential to predict the survival and immunotherapy response of BLCA patients. In these studies, the CAFs-related risk genes were selected based on the subtyping of CAFs. Unlike these studies, we investigated the CAFs-related risk signatures from the perspective of circadian rhythm regulation. In this research, a risk model was established with genes implicated in the activation signals of fibroblasts within the circadian-related subgroups. This risk model effectively discriminated BLCA tumors with varying degrees of CAFs infiltration and distinct TME profiles. Firstly, the GSEA analysis revealed high enrichment of genes implicated in fibroblasts, endothelial cells, as well as TME constitutions in the high-risk group. Secondly, three CAFs-containing algorithms (EPIC, XCELL, and MCPCounter) revealed higher CAFs infiltration in the high-risk tumors. Thirdly, the TIDE score indicated augmented T cell exclusion immune phenotype in the high-risk group, which is mainly favored by the CAFs [31]. Therefore, the risk model is associated with CAFs infiltration in BLCA tumors. Consequently, the risk model can predict the efficacy of BLCA immunotherapy in the IMvigor210 cohort. High-risk patients exhibited significantly worse overall survival compared to low-risk patients. These findings suggest that the risk model is associated with CAFs abundance and holds promise for predicting the efficacy of BLCA immunotherapy. Additionally, the risk model was associated with sensitivity to chemotherapy drugs such as oxaliplatin, gemcitabine, and cytarabine, among others. Currently, neoadjuvant cisplatin-based chemotherapy is recommended for eligible patients with muscle-invasive bladder cancer [51]. However, a subset of patients does not benefit from this neoadjuvant chemotherapy [52]. Considering the pivotal role of CAFs in chemotherapy, this fibroblasts-based risk model may be utilized to more effectively stratify patients who will benefit from chemotherapy.

To further explore the regulatory nexus between the risk genes and the circadian rhythm, we conducted a correlation analysis between the risk genes and the CCRGs, in the TCGA-BLCA, GSE13507, and GSE32894 datasets. As expected, the expression of the risk genes exhibited broad correlations with CCRGs across the three datasets. Among the five risk genes, *FAM20C* and *TBX1* were found to have the most extensive correlations with the CCRGs. What’s more, their correlations with CCRGs were diametrically opposite, suggesting the existence of a regulatory network involving FAM20C, TBX1, and CCRGs. Furthermore, we demonstrated that *FAM20C* and *TBX1* are associated with CAFs infiltration in contrasting manners, as *FAM20C* exhibited a positive correlation while *TBX1* showed a negative correlation with CAFs. This highlights the predictive value of these two genes in the context of immunotherapy. Ultimately, our findings revealed that *FAM20C* can predict the efficacy of BLCA immunotherapy, whereas regrettably, *TBX1* failed to serve as a predictor of immunotherapy efficacy.

FAM20C is a member of the Fam20 family known for its association with Raine Syndrome [53]. It assumes a pivotal role in phosphorylating numerous secreted proteins and multiple substrates, contributing to diverse biological functions [54]. The best characterized function of FAM20C is its kinase activity on mineralization proteins, such as the fibroblast growth factor 23 (FGF23) [54]. FAM20C can phosphorylate FGF23 post-translationally, leading it to FURIN (PCSK3)-dependent proteolysis [55]. Beyond its involvement in mineralization, FAM20C participates in other processes across multiple organs, including wound healing, lipid homeostasis, adhesion and cell migration [54]. In cancer context, FAM20C has been implicated in enhancing the metastasis of several human cancers, making it a potential therapeutic target [56]. Notably, some small molecule inhibitors have already been reported in triple-negative breast cancer [57]. A recent study further revealed that FAM20C mediates the invasive growth of stem-like cells in glioblastoma [58]. Although FAM20C is well recognized as a pro-malignant factor, its association with immunotherapy has not been previously documented.

In this study, *FAM20C* was identified as an independent predictor for survival, with high *FAM20C* expression correlating with poor overall survival. This detrimental effect of FAM20C may be attributed to its promotional impact on CAFs, supported by several reasons: (1) FAM20C gene expression exhibited a robust correlation with CAFs infiltration. (2) High FAM20C expression, combined with high CAF levels, resulted in the poorest survival outcomes. (3) The survival disadvantage associated with FAM20C was mitigated by low CAF levels. Our findings suggest a collaborative pattern between FAM20C and CAFs that detrimentally impacts survival. Supporting this notion, FAM20C displayed potential in predicting immunotherapy efficacy. Taken together, these results underscore the promoting effect of FAM20C on cancer malignant progression and indicate a collaborative pattern between FAM20C and CAFs that warrants further exploration.

In conclusion, disruption of the circadian rhythm may impact the effectiveness of both chemotherapy and immunotherapy by bolstering CAFs activation. This study has identified a circadian-based risk signature for evaluating CAFs infiltration and forecasting the efficacy of chemotherapy and immunotherapy. Moreover, FAM20C emerged as a circadian-related gene with prognostic significance for BLCA immunotherapy. In addition, glycosaminoglycan and chondroitin sulfate metabolism, certain oncogenic genes (*mTOR, YAP, LEF1*, and *CYCLIN D1*), and specific transcription factors (*AP1, ATOH8, TEF1*, and *NFE2*) were notably enriched in the high-risk tumors. Comprehending these intricate interconnections will yield valuable insights for potential therapeutic approaches aimed at counteracting CAFs and ultimately improving cancer treatment outcomes.

## Supplementary Material

Supplementary Figures
