# Supplementary material for: Identification of a circadian-based prognostic signature predicting cancer-associated fibroblasts infiltration and immunotherapy response in bladder cancer
Source: Aging (Albany NY). 2024 Aug 30;16(17):12312–34. doi: 10.18632/aging.206088 (PMC11424586; doi:10.18632/aging.206088)
Supplement: Supplementary Figures [file aging-16-206088-s001.pdf]

SUPPLEMENTARY FIGURES

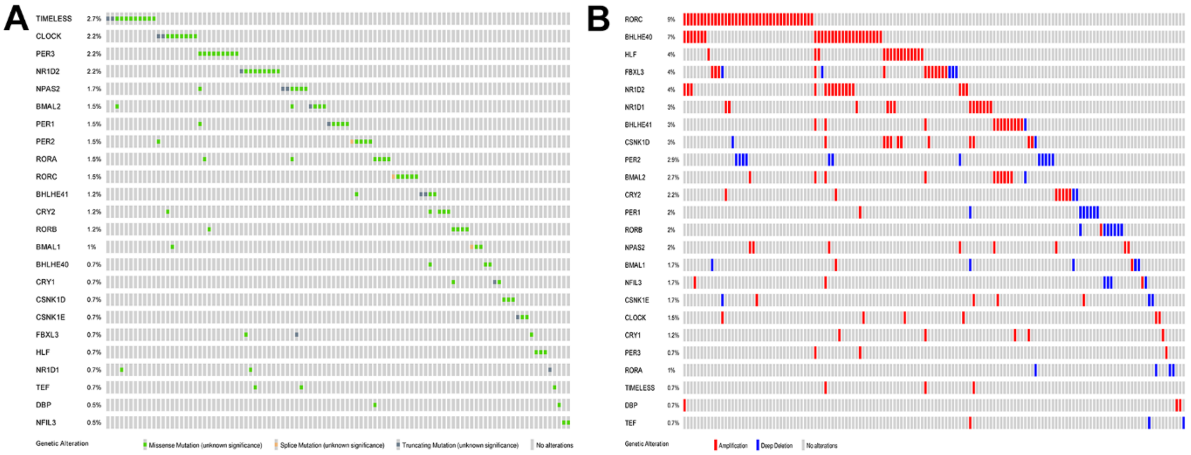

Supplementary Figure 1. Genetic alterations of the TCGA-BLCA. (**A**) Genetic mutations and (**B**) the copy number variation (CNV) of the 24 CCRGs were analyzed through the cBioPortal websites.

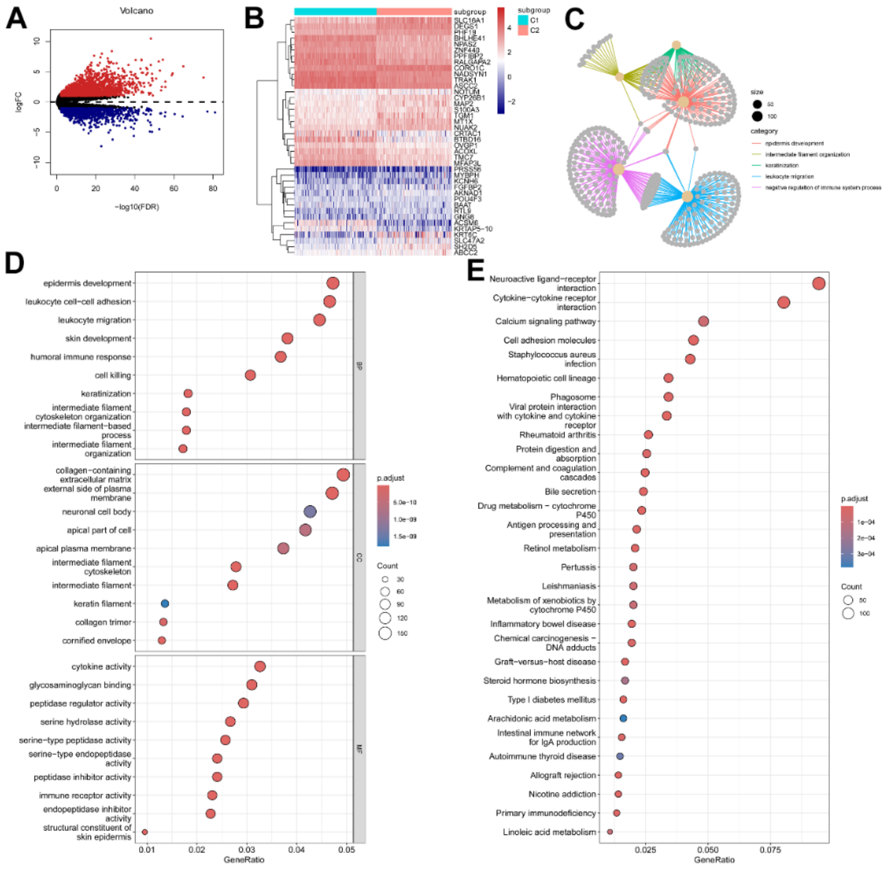

Supplementary Figure 2. DEGs between the circadian rhythm-based subgroups. (**A**) Volcano plot of the DEGs between the C1 and C2 circadian-based clusters. (**B**) Heatmap plot of the top 40 DEGs. (**C**) Enrich-plot and (**D**) bubble charts of the GO enrichment terms of the DEGs. (**E**) Bubble charts of the KEGG enrichment terms.

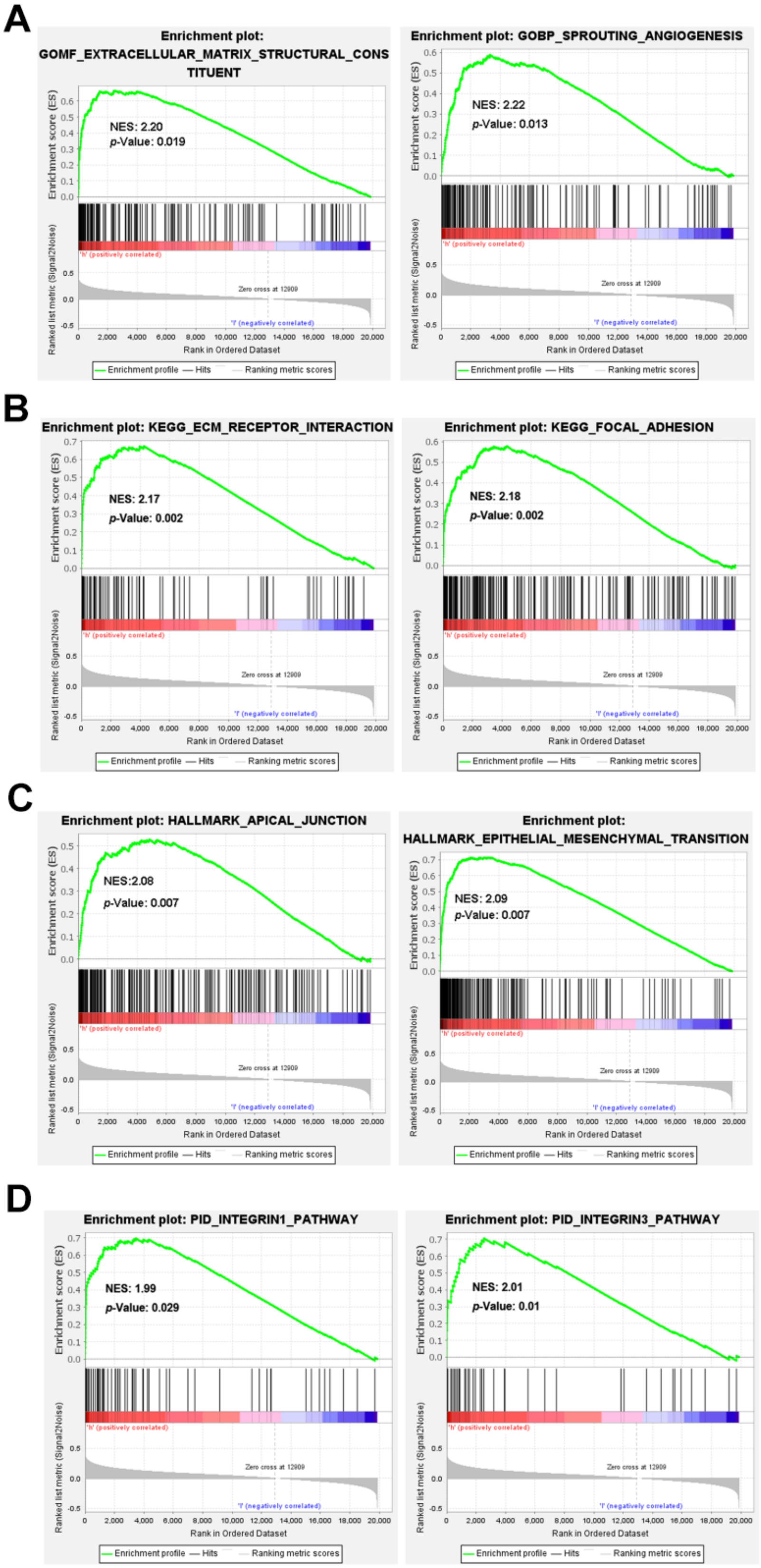

Supplementary Figure 3. GSEA enrichment between high- and low-risk groups. GSEA enrichment based on (**A**) the GO, (**B**) KEGG, (**C**) HALLMARK, and (**D**) the PID (pathway interaction database) gene sets.

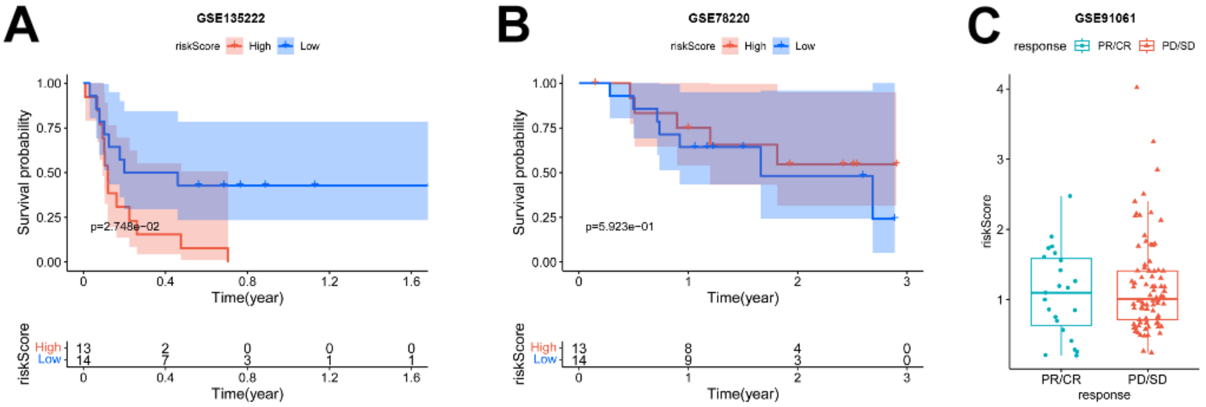

Supplementary Figure 4. Different immunotherapy response between high- and low-risk groups. Kaplan-Meier curve of the high- and low-risk patients in tumor immunotherapy cohorts of (**A**) non-small cell lung carcinoma (GSE135222), and (**B**) melanoma (GSE78220). (**C**) Relationship of risk score with immunotherapy response in melanoma cohort GSE91061.

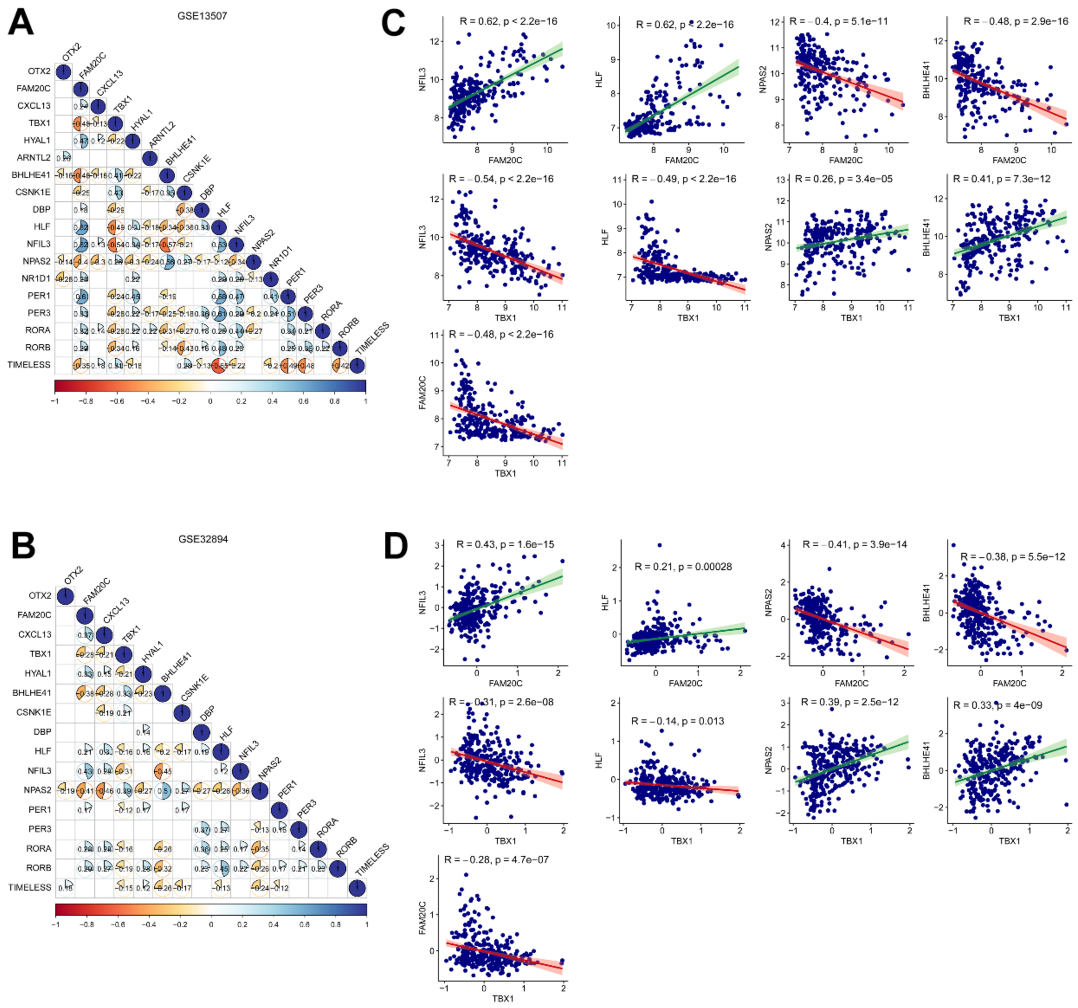

Supplementary Figure 5. Correlated expression of the risk genes and the CCRDs. (**A**, **B**) Corrplot of the correlated expression of the risk genes with the CCRGs in GSE13507 and GSE32894 datasets. (**C**, **D**) Correlation of the indicative genes in GSE13507 and GSE32894 datasets. Method = "Pearson".
